# Viral Nucleases

**DOI:** 10.3390/v15030740

**Published:** 2023-03-13

**Authors:** Zhengqiang Wang, Robert J. Geraghty

**Affiliations:** Center for Drug Design, College of Pharmacy, University of Minnesota, Minneapolis, MN 55455, USA

Nucleases are ubiquitous hydrolytic enzymes that cleave phosphodiester bond of DNA (DNases), RNA (RNases), or protein-RNA/DNA (phosphodiesterases), within the strand (endonucleases) or from the end (exonucleases) [1]. Diverse active site structures and catalytic mechanisms of viral nucleases enable a wide range of essential roles for viruses. Notable viral nuclease functions include genome replication (RNase H [2] of HIV and HBV), cap snatching [3,4] (endonucleases of influenza virus, bunyavirus, and arenavirus), genome packaging [5] (herpesvirus terminase endonuclease), proof reading (SARS-CoV-2 NSP14 ExoN [6]), genome repair (phosphodiesterases of picornaviruses [7] and HBV [8]), genome recombination (Holliday junction resolvase [9] of orthopoxvirus), and innate immune evasion (SARS-CoV-2 NSP15 EndoU [10]). Many of these nuclease functions have been targeted by medicinal chemistry and drug discovery research [11,12,13], and the successful development of the PA endonuclease-targeting baloxavir [14] as an influenza drug has provided clinical validation for nuclease-targeting antiviral research. However, the structures, functions, catalytic mechanisms, and druggability of most viral nucleases remain underexplored. This Special Issue, entitled “Viral Nucleases”, provides a snapshot of nuclease-targeting antiviral research, with a strong and timely focus on SARS-CoV-2 proofreading exonuclease (ExoN). Unlike most RNA viruses that lack a proofreading mechanism, coronaviruses encode a unique 3′ to 5′ ExoN to proofread and maintain their relatively large RNA genomes. This function also creates a barrier for anti-coronaviral nucleoside analogs as incorporated analogs can be excised by ExoN. Inhibiting ExoN could lead to two distinct pharmacological effects against CoV: conferring antiviral phenotypes by promoting lethal mutagenesis and enhancing antiviral efficacy of nucleoside analogs by reducing excision.

The report by Ju and co-workers (https://doi.org/10.3390/v14071413 (accessed on 7 March 2023)) studied nucleotide structural features that conferred resistance to ExoN excision. Significantly, it was observed that 2′-deoxynucleotides with or without the 3′-OH were resistant to ExoN excision, whereas analogs with both 2′- and 3′-OH were efficiently removed. Another observation was that 3′-OH was more important than 2′-OH for ExoN excision. These findings provide insights into the design of nucleos(t)ide RdRp inhibitors that are resistant to ExoN excision.

Aihara and co-workers reported that ExoN excision of natural antiviral chain-terminating nucleotide ddhCMP (https://doi.org/10.3390/v14081790 (accessed on 7 March 2023)) incorporated by RdRp was more efficient than 3′-dCMP. Interestingly, this trend was reversed in incorporation where 3′-dCTP was more efficiently incorporated than ddhCTP. These observations indicate a possible role of nsp14 ExoN in protecting SARS-CoV-2 from ddhCTP, a product of innate immune antiviral response.

In a report by Chaves et al., seven flavonoids were tested in cell-based antiviral assays against SARS-CoV-2 (https://doi.org/10.3390/v14071458 (accessed on 7 March 2023)). Their studies revealed the impact of increasing the number of hydroxyl groups on antiviral potency. In vitro enzymatic assays and in silico molecular docking suggest that these compounds may dually inhibit both ExoN and Mpro.

This Special Issue also features a comprehensive review from Weller and co-workers on genome recombination and/or proofreading nucleases of two hugely different viral classes, human herpesviruses (HHVs) and coronaviruses (CoVs) (https://doi.org/10.3390/v14071557 (accessed on 7 March 2023)). The review focuses on the virological roles of two HHV exonucleases (alkaline nuclease and PolExo) and one CoV exonuclease (ExoN), and provides valuable discussions on chemotypes, particularly metal-binding compounds, for their inhibition.

Collectively, these papers highlight the significance of antiviral research targeting viral nucleases and could stimulate further interest in this area of research.
